# The Standard Operating Procedures in COVID-19 Pandemic for Periodontal Aerosol-Generating Procedures: A Process Audit

**DOI:** 10.1055/s-0042-1758067

**Published:** 2022-12-30

**Authors:** Muhammad Haseeb, Naima Khalid, Azeem Ul Yaqin Syed, Zubair Ahmed Khan, Farheen Qureshi, Iftikhar Ahsen

**Affiliations:** 1 Department of Periodontology, University College of Medicine and Dentistry, The University of Lahore, Lahore, Pakistan; 2 Department of Periodontology, FMH College of Medicine and Dentistry Lahore, Lahore, Pakistan; 3 Department of Prosthodontics, College of Dentistry, University of Science and Technology of Fujairah, Fujairah, United Arab Emirates

**Keywords:** aerosol-generating procedures, clinical audit, COVID-19, standard operating procedures, ultrasonic scaling

## Abstract

**Objective** This study aims to audit the process of patient management with aerosol-generating procedure (ultrasonic scaling) while adherence to the guidelines for health care workers (HCWs) during the coronavirus disease 2019 (COVID-19).

**Materials and Methods** Audits records at the Department of Periodontology at University College of Medicine and Dentistry Hospital, Lahore, Pakistan were collected (prospectively) over the period of October 1 to November 30, 2020 (1st cycle) and December 14, 2020 to February 12, 2021 (2nd cycle). The audit was divided into three components based on the guidelines: (1) physical environment, (2) patients/appointments, and (3) COVID standard operating procedures related to HCWs.

**Results** The recommended physical layout and procedural factors, as suggested by the guidelines for dental clinics, were observed during the first cycle of audit, and discrepancy of ventilation system was fixed after the first cycle. Audit team reported the observance of fallow time three times daily, which revealed 83.3% observance of fallow time.

Later in the second cycle when the extraoral high-volume air evacuator was installed, the fallow time was reduced to 15 minutes and not only five procedural slots per day were created but fallow time was also observed 100% of the time.

**Conclusion** Following the standard guidelines resulted in more efficient working environment and lesser risk for HCWs while performing aerosol-generating procedures.

## Introduction

Coronavirus disease 2019 (COVID-19) is a major worldwide public health concern that has had a significant health and socioeconomic impact till date. The influence of the COVID caused by the single-stranded ribonucleic acid virus severe acute respiratory syndrome coronavirus 2 (SARS-CoV-2) has had a significant impact on daily life, with almost a third of the world's population under a state of “lockdown” as the globe struggles to contain the pandemic. 1 2

As the COVID virus spread, quarantine and social isolation were encouraged to limit and prevent the disease's spread. More stringent rules in hospitals were implemented to prevent nosocomial infection, including visitors to wear face masks before entering, measuring body temperature, and collecting all visitors' travel, occupation, contact, and cluster histories, limiting the number of people who accompany patients, and adhering to the triage and workflow decorum for suspected nosocomial infections. 3

The severity of the COVID-19 pandemic has drastically altered the dental practice perspective in terms of operating procedures, treatment, and infection control protocols. COVID had a substantial influence on dentistry due to virus' aerosol transmission. As a result, dental professionals are at a significant risk of getting the virus and distributing it to the public through aerosol-generating procedures (AGPs). 4 In routine dental treatment, aerosols commonly combine with saliva, increasing the risk of COVID transmission through the air. Varied global and local professional bodies in dentistry have proposed various guidelines, with varying levels of response depending on the prevalence of COVID in distinct places, countries, and regions. 5 Government orders issued on March 25, 2020, instructed dental care practices to discontinue all nonurgent dental treatment indefinitely. In accordance with the instructions of the local government, infection control recommendations have focused on reducing the possible risk of disease contamination and dissemination in dental practice. Patients are still in a state of flux, as primary care clinics have been directed to provide telephone triage and, if practicable, to use the “three A's” strategy of providing advice, treating with analgesics, and antimicrobial prescription. Structural audits and process audits can provide policymakers with a solid foundation for developing strategies and structured plans for dealing with the pandemic in dental facilities. 6

**Fig. 1:**
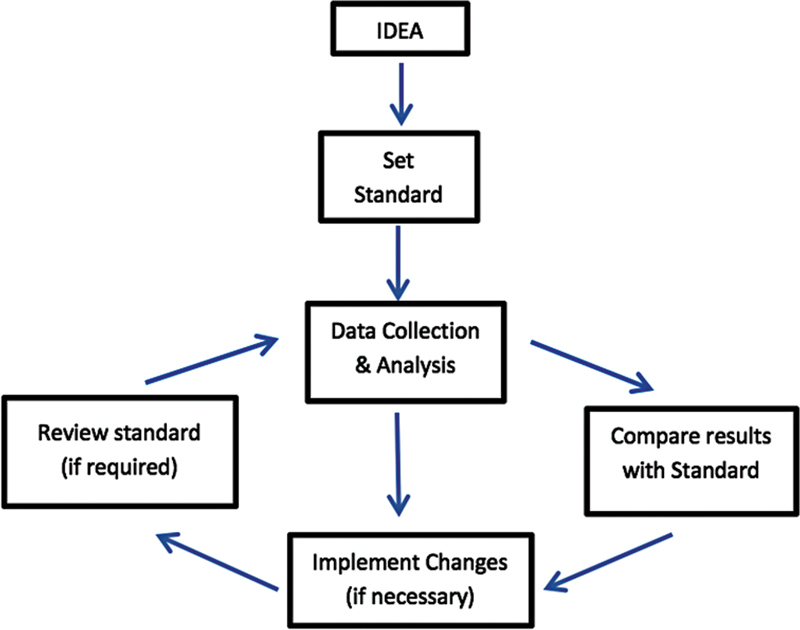
The audit process.

Resuming dental procedures especially the AGPs need to be audited so that risk of transmission can be assessed and necessary measure taken to reduce it. Clinical audit should be the method used by all health care professionals to assess, evaluate, and then improve the treatment of patients in a systematic manner in order to improve their health and quality of life. 7 The process consists of four basic steps that are shown in Fig. 1 . Auditing has been found to be effective in the dental setting, where the notions of critical review and reflective practice are emphasized. Therefore, dental practitioners and departmental heads are encouraged to complete an audit project. 8

Given the scarcity of data on the adherence of dental personnel and institution to COVID-19 guidelines and protocols in Pakistan, this study primarily aimed to audit the process of patient management with AGP (ultrasonic scaling) while adherence to the guidelines for health care workers (HCWs) during the COVID-19.

## Materials and Methods

The Department of Periodontology is a 16-dental unit department in the University Dental Hospital (UDH) at the University of Lahore catering to an average of 600 patients monthly. The Periodontology Department employs 11 full-time employees including 2 assistant professor, 5 demonstrators, and 4 dental assistants whereas an average of 12 house officers come on two monthly rotations.

In the year 2020, all dental departments were forced shut from March 26 till September 15 and after necessary changes the Department of Periodontology started functioning from October 1, 2020. For AGPs in the Department of Periodontology, procedural slots were created each day. In each cycle, four procedural sots per day were introduced with the fallow time of 30 minutes in between each procedural slot.

Audit records of the Periodontology Department of UDH were collected over the period of October 1 to November 30, 2020 (1st cycle) and December 14, 2020 to February 12, 2021 (2nd cycle). A series of daily audits was conducted for 16 weeks. All faculty members, paramedics, and house officers of the Periodontology Department were informed about the prospective nature of data collection by means of a circular distributed in September 2020.

Over the course of several discussions with the focus group of six faculty members, the audit tool was devised, and content validation was done. The audit items were chosen after a review of standards and guidelines from the Public Health England COVID-19 Infection Prevention and Control Dental Appendix, Scottish Dental Clinical Effectiveness Programme Mitigation of Aerosol-Generating Procedures in Dentistry, and Government of Pakistan guidelines for return to work guidance for providing dental care services in COVID-19. The audit tool was shared with the hospital management, and they were asked for input. Prior to the audit, the permission was granted from the Ethical Research Committee of University College of Medicine and Dentistry. To ensure fairness and objectivity, it was emphasized that anonymity will be respected and that a standardized audit form will be utilized during the inspection and review process. The review team involved two faculty members of the department and two members of the hospital administration. The parameters were analyzed daily at midday and recorded on the audit form especially designed for this purpose.

The audit was divided into three components based on the guidelines: (1) physical environment and procedural parameters, (2) patients/appointments, and (3) COVID standard operating procedures (SOPs) related to HCWs. The audit report for patient's appointment was collected each day whereas audit of SOPs related to HCW were collected once a week by a different examiner from October 1 to November 30, 2020 (1st cycle). Each auditor presented the audit findings and highlighted relevant concerns and opportunities for improvement, followed by interventions (installation of external ventilators) and implementations of COVID-19 SOPs. Then, the second cycle of audit was conducted from December 14, 2020 to February 12, 2021.

Inspection of the physical environment including the procedural parameters consists of a general examination of the layout of the units and the area at one point of time for each cycle. However, COVID SOPs related to HCWs and patient's factors were observed and documented once every week and once daily during both the cycles, respectively.

## Results

The results were categorized into two parts: (1) outcome of the first cycle audit observations and (2) outcome of the second cycle.

Table 1 shows the results of observations conducted for physical layout and procedural considerations throughout the two cycles of audit. After the first audit, an intervention plan was discussed and executed. After intervention, audit session was carried out for the next 2 months. The recommended physical layout and procedural factors, as suggested by the guidelines for dental clinics, were observed during the first cycle of audit, and discrepancy of ventilation system was fixed.

**Table 1:** Physical environment parameters a

**Items**	**Cycle 1** **(October 2020–November 2020)**	**Intervention (December 1–December 12, 2020)**	**Cycle 2** **(Mid December 2020–Mid February 2021)**
Is the area naturally or mechanically well ventilated?	No		Yes
Is there any special area designated for removal of PPEs?	Yes		Yes
Is adequate distance maintained between dental operatories?	Yes		Yes
Are high-volume suctions available?	Yes		Yes

Abbreviation: PPE, personal protective equipment.

a Observations were recorded once per audit cycle.

Only one house officer got COVID positive on 3rd day of house job. Contact tracing of all the colleagues were done and polymerase chain reaction (PCR) test was advised. A total of nine contacts were traced which were tested negative ( Table 2 ).

**Table 2:** COVID SOPs related to HCWs a

**Items**	**Cycle 1** **(October 2020–November 2020)**	**Intervention (December 1–December 12, 2020)**	**Cycle 2** **(Mid December 2020–Mid February 2021)**
Was dissemination of knowledge to the faculty, paramedics and students carried out regarding mitigation of AGPs in COVID'19?	Weekly		Yes
Does any incident of COVID positive health care worker reported?	Yes (First week)		No
Was contact tracing done after any COVID positive incident?	Yes		Not applicable

Abbreviations: AGP, aerosol-generating procedure; COVID-19, coronavirus disease 2019; HCW, health care worker; SOP, standard operating procedure.

a Observations were recorded weekly during both audit cycles.

There were four procedural slots per day with three fallow time of 1 hour each in the first cycle. Audit team reported the observance of fallow time three times daily, which was 83.3% of the times. There were few incidents (17.7%) of breach of fallow time since there was no artificial ventilation system and 1 hour of fallow time was observed, which in few instances was difficult to maintain.

Patients reporting in dental outpatient department (OPD) who were referred for scaling procedure were put on the wait list after examination, which ranged between 1 week and up to 4 weeks because of the four procedural slots per day. These patients were given appointments and were strictly asked to report on time. In case of any dropouts, patients reporting in OPD were given chance. In most of the cases, waiting time to get the procedure done was more than 2 weeks ( Table 3 )

**Table 3:** Patients/Appointments a

**Items**	**Cycle 1** **(October 2020–November 2020)**	**Intervention (December 1–December 12, 2020)**	**Cycle 2** **(Mid December 2020–Mid February 2021)**
Was fallow time observed after every cycle of AGPs?	83.3%		100%
How many appointed patients had to wait > 10 minutes before the start of their procedure?	28.2%		15.3%
How many patients were put on the wait list (> 2 weeks between patient OPD and procedure)?	74.8%		61.9%
Do patients undergo screening test for COVID before AGPs?	No		No

Abbreviations: AGP, aerosol-generating procedure; COVID-19, coronavirus disease 2019; OPD, outpatient department.

a Observations were recoded daily during both audit cycles.

Once the patient reported at the department on the appointed day, dental staff was advised not to make them wait to avoid crowding in the waiting room. There were few occasions in each cycle where the appointed patients had to wait more than 10 minutes before they were seated, and waiting time decreased in the second cycle when the dental staff was sensitized with its importance repeatedly as part of our weekly dissemination of knowledge.

## Discussion

Lockdown period provided a necessary time frame for the stakeholders to work on guidelines and SOPs before they could resume their services. Among the factors to consider were facilities, human resources, and personal protective equipment stock. On these bases, UDH developed institutional guidelines, which were consistent with the national guidelines and recommendations (Government of Pakistan guidelines for return to work guidance for providing dental care services in COVID-19). 9

The success of guidelines depends on how it is translated into clinical work. Clinical guidelines are developed from a series of evidence-based literature; thus, compliance to guidelines may result in good clinical outcomes, especially during a pandemic. In this audit report, we observed the environmental infrastructure and implementation of dental staff and students to COVID SOPs recommendations and facility preparedness in facing the new norm within the Department of Periodontology. The primary dental procedures observed in the Periodontology Department is ultrasonic scaling that generate splatter, droplets, and aerosols of a range of particle sizes and thus is one of the main AGPs.

During the first cycle, we realized that fallow time was not being implemented because the Periodontology Department was not naturally or mechanically ventilated for AGPs. In the intervention period, extraoral high-volume air evacuators were installed in the department to create artificial or mechanical ventilation and significant improvements were seen with respect to risk categorization due to reduced fallow time (the time required to allow larger droplets to settle before environmental cleaning) to 15 minutes. 10

A pragmatic fallow time is recommended to reduce the potential risk of SARS-CoV-2 transmission associated with treatment that involves AGPs. The purpose of fallow time is to allow aerosols to get settled. It incorporates an assumption that high-volume suction is a standard practice for most AGPs. A benchmark fallow time, dictated by ventilation rate, is of 15 to 30 minutes; but when ventilation is poor and suction is not used, this time is longer (up to 60 minutes). Initially, there were many other techniques attempted to purify the air, but as the evidence gathered the use of air cleaners were not recommended to reduce the potential risk of SARS-CoV-2 transmission associated with dental AGPs. 11

In settings where multiple chairs are used in the same room and AGPs are performed there needs to be a physical spacing of at least 2 m and a method of physical segregation (dividers) that provides at least a 2-m barrier in the horizontal and vertical plains. 12 Ideally, each “pod” should have adequate ventilation. Prior to COVID-19 pandemic, we had eight dental units (without vertical dividers) in the AGP room out which we only used four dental units at a time, keeping more than 2 m distance.

A designated entrance with proper screening triage with the appointed staff members were set up to control incoming patients to hospital OPD. Patient's relatives were not allowed unless the patient is dependent. All who enter were screened for body temperature and provided hand sanitizers. Amidst the plethora of guidelines, there was evidence that patients getting AGP should be tested with PCR within 72 hours. This could not be exercised in our setting in both the cycles due to financial constraints. 13 Furthermore, departmental data was being monitored frequently and no transmission among HCW encouraged sticking to the current practices. Patients were dually informed about the wait list since we were catering the patients in slots to decrease the transmission risk. Wait list decreased in the second cycle because we had five slots per day but still majority patients had to wait more than 2 weeks. Prior to the lockdown the wait list was few days only. Attempt to minimize the crowding in the waiting area was again diligently done by sensitizing dental assistants, dental staff, and putting on paper stickers so that patients maintain the adequate distance. Patients were asked to come 15 minutes before their appointment time so that they can complete the payments and then procedural entry in the system. We considered a margin of 10 minutes to complete the abovementioned tasks, above which it was recorded extra wait time in the audits.

When ventilation is inadequate (e.g., 1–2 air changes/hour [ACH]), high-volume suction is considered necessary. If this is not practicable, a 60-minute fallow period might be considered. Likewise, when ventilation is minimal (1–5 ACH) or uncertain (ventilation is present but the number of changes is unknown), a 30-minute fallow period is recommended. 11 The air changes before and after the installation of the air evacuators were not recorded in our investigation, which was the limitation of our study.

Process auditing can be a daunting endeavor. A standardized audit methodology serves as a template for an unbiased, systematic, structured, and complete evaluation. Uniformity of approach, involvement of the clinical area under review, and documenting outcomes has been beneficial in establishing and enhancing COVID-19 control protocol standards in our institution.

## Conclusion

A standardized protocol for the audit process provides a template for an impartial, organized, structured, and thorough review. The uniformity of approach, engagement of the clinical area under evaluation, and documenting of results have all been beneficial in promoting and strengthening COVID protocol standards in this institution. Guidelines and briefings provided to employees and students aided in providing knowledge into the necessity of COVID-19 prevention and control. Being aware and knowing what to do could help to lessen the impact of the pandemic on the dentistry.
